# Decoding microbial genomes to understand their functional roles in human complex diseases

**DOI:** 10.1002/imt2.14

**Published:** 2022-03-29

**Authors:** Yifeng Wang, Quanbin Dong, Shixian Hu, Huayiyang Zou, Tingting Wu, Jing Shi, Haifeng Zhang, Yanhui Sheng, Wei Sun, Xiangqing Kong, Lianmin Chen

**Affiliations:** ^1^ Department of Cardiology, The First Affiliated Hospital of Nanjing Medical University Nanjing Medical University Nanjing Jiangsu China; ^2^ Cardiovascular Research Center, The Affiliated Suzhou Hospital of Nanjing Medical University, Suzhou Municipal Hospital, Gusu School Nanjing Medical University Suzhou Jiangsu China; ^3^ Institute of Precision Medicine, The First Affiliated Hospital of Sun Yat‐Sen University Sun Yat‐Sen University Guangzhou Guangdong China; ^4^ Department of Genetics, University Medical Center Groningen University of Groningen Groningen The Netherlands

**Keywords:** gut microbiome, metabolites, complex disease, host–microbe interactions

## Abstract

Complex diseases such as cardiovascular disease (CVD), obesity, inflammatory bowel disease (IBD), kidney disease, type 2 diabetes (T2D), and cancer have become a major burden to public health and affect more than 20% of the population worldwide. The etiology of complex diseases is not yet clear, but they are traditionally thought to be caused by genetics and environmental factors (e.g., dietary habits), and by their interactions. Besides this, increasing pieces of evidence now highlight that the intestinal microbiota may contribute substantially to the health and disease of the human host via their metabolic molecules. Therefore, decoding the microbial genomes has been an important strategy to shed light on their functional potential. In this review, we summarize the roles of the gut microbiome in complex diseases from its functional perspective. We further introduce artificial tools in decoding microbial genomes to profile their functionalities. Finally, state‐of‐the‐art techniques have been highlighted which may contribute to a mechanistic understanding of the gut microbiome in human complex diseases and promote the development of the gut microbiome‐based personalized medicine.

## Highlights

- Functional roles of the gut microbiome in human health and disease.
- Commonly used tools in decoding microbial functionalities.
- State of the art techniques in validating microbial functionalities.

## Graphical abstract

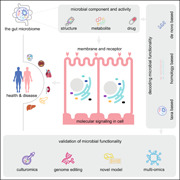

## INTRODUCTION

Genome‐wide association studies (GWAS) have dissected the genetic architecture of human complex diseases, which has advanced our understanding of disease etiology and promoted the development of genome‐based therapy [1]. However, genetics can only explain a limited proportion of an individual's risk of developing a complex disease [2]. For instance, GWAS can only explain the heritability of type 2 diabetes (T2D) and Crohn's disease with 6% and 20% [2] success, respectively. Recently, the contribution of the gut microbiome to the development of complex human diseases has increasingly been recognized ‐ and become a booming field of research [3, 4, 5, 6, 7, 8, 9, 10, 11].

The human intestines are colonized by a vast number of bacteria, archaea, microbial eukaryotes, and viruses, as abundant as our somatic cells, which are collectively known as the gut microbiome [12]. The gut microbiome has been involved in digesting food, training host immunity, regulating gut endocrine function and neurological signaling, modifying drug action and metabolism, eliminating toxins, and producing numerous compounds that influence the host [13]. In mice studies, gut microbiota has been shown to be essential for germ‐free animal models to develop inflammatory bowel disease (IBD) [14]. Human infants born from mothers with immune‐related diseases presented altered gut microbial compositions which was further proved to have the potential to trigger adaptive immune response [15, 16, 17]. All the evidence pinpoints to the critical roles of gut microbiota in developing complex diseases.

Rapid development of metagenomics sequencing technology and big cohort studies allow us to integrate gut microbiome profiles with host clinical phenotypes, to identify candidate disease‐related microbial features in a large scale. Numerous associations between the gut microbial composition and complex diseases have been reported, including but not limited to cardiovascular disease (CVD), diabetes, IBD, allergy, and cancer [3, 4, 5, 6, 7, 8, 9, 10]. Unlike the human genome, modification of gut microbial communities is feasible and ethical, the gut microbiome is thereby emerging as an attractive therapeutic target for disease prevention and treatment. However, there are still big gaps between research and clinical translation, including the lacking consistency of disease‐specific microbial taxa across studies, poor causal inference, and unsatisfactory efficiency of current microbiome‐based therapies in patients (e.g., fecal microbiome transplant and probiotic usage). These could be as a result of (1) many gut bacteria are opportunistic, and they could present adverse effects differently dependent on specific conditions, (2) most of studies focus on the microbial composition which is far from enough because different subspecies could behave differently. Functional analysis by decoding the microbial genomes found that microbial genes like *cutC/D* are responsible for the biosynthesis of phenylacetylglutamine and trimethylamine‐*N*‐oxide (TMAO), two metabolites that can induce CVD risk [18, 19]. Therefore, going beyond microbial composition and understanding the gut microbial functionalities could facilitate to shed light on the issues above.

Here, we summarize recent research advances in the intestinal microbiome related to human health and disease, with a particular focus on their functionalities, mainly including microbial virulence factors such as capsule and biofilm, microbiota‐derived small molecules, and drug metabolize. We further introduce artificial tools in decoding microbial genomes to characterize the functional potential. Finally, we highlight state‐of‐the‐art techniques that may help us gain a mechanistic understanding of the gut microbiome in human complex disease and to promote the development of gut microbiome‐based personalized medicine.

## MICROBIAL FUNCTIONALITIES THAT AFFECT HUMAN HEALTH AND DISEASE

### Via specific structures

**Figure 1:**
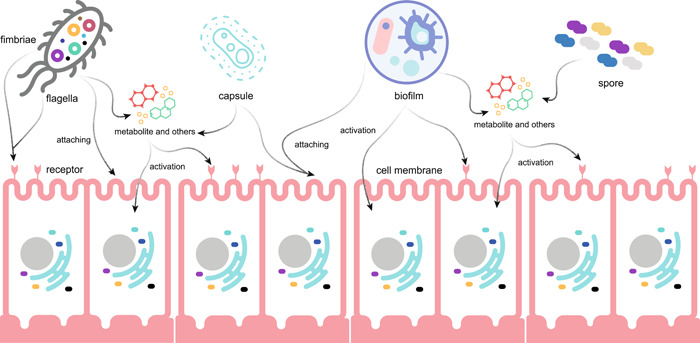
Microbial structures that contribute to resistance and virulence. The direct influence of gut microbes on the host can be attributed to the fundamental structures that lead to resistance and virulence such as flagella and fimbriae, capsule, spore, and biofilm, which facilitate the survival and activity of trillions of bacteria in the human intestine. Specific microbial structures can help microbes attach human cells or receptors which further activates various signaling pathways. Besides this, they may carry antibiotic resistance and virulence factors, as well as various metabolites and other disease‐relevant molecules

The direct influence of gut microbes on the host can be attributed to the fundamental structures that lead to resistance and virulence such as flagella and fimbriae, capsule, spore, and biofilm, which facilitate the survival and activity of trillions of bacteria in the human intestine [20, 21] (Figure 1).

Fimbriae are straight filaments arising from the bacterial cell wall while flagella are much longer than fimbriae. Flagella spins the spirochete around and generates thrust, propelling bacteria moving forward. The formation of fimbriae and flagella always relies on gene clusters and varies substantially between species. For instance, the *Salmonella fim* cluster comprises 10 genes [22] while in *Escherichia coli* more than six gene clusters for fimbriae formation are identified [23]. Both can lead to host infection, but the mechanisms are different. Many bacterial pathogens require motility to infect, including *E. coli*, *Salmonella enterica* and others [24], thus flagella play key roles during this progress. Unlike flagella, fimbriae carry virulence factors and help in the adherence of bacteria to human cells. For instance, *Bordetella pertussis* uses its adhesin to bind to ciliated respiratory cells and cause whooping cough [25]. Fimbriae of *Neisseria gonorrhea* help it to bind to cervical cells and buccal cells to cause gonorrhea [26]. Without fimbriae to bind to the intestinal epithelium, *E. coli* and *Campylobacter jejuni* cannot cause diarrhea [27].

Capsule is a polysaccharide layer that lies outside the cell envelope and is considered a part of the outer envelope of a bacterial cell. The capsule is found in both Gram‐negative and positive bacteria. However, it is different from the second lipid membrane, which contains lipopolysaccharide (LPSs) and lipoproteins found only in Gram‐negative bacteria [20]. The capsule protects bacteria from mechanical injury and environmental changes (such as temperature, drying, bacteriophages, and eukaryotic cells) [28]. It also helps in the adherence of bacteria to smooth surfaces. For example, *Streptococcus mutans*, which causes dental caries, attaches to the surface of the teeth by its capsule [29]. The capsule is essential for pathogenic microorganisms to invade the host immune system and prevents them from being phagocytosed by macrophages and neutrophils [30]. A study revealed that the thickness of the capsule in *Streptococcus pneumoniae* was associated with the severity of meningitis [31]. Interacting with β‐glucans on the fungal cell wall during fungi infection leads to host Dectin‐1‐related CARD9 signaling pathways activation which can induce inflammation [32]. However, capsular materials have also been successfully used as vaccination against *S. pneumoniae* and *Haemophilus influenza* [33].

Unlike capsule, the spore is a very hardy cell and allows a bacterial cell to survive under even the worst conditions. Therefore, spore can protect the pathogenic bacteria from antibiotics and other injures to produce virulence factors [34]. *Bacillus* and *Clostridium* species are the most common bacteria to create spores and can induce various infection diseases [34]. For example, *Bacillus cereus* is well‐known for its ability to cause foodborne illness because of its spores surviving various temperatures [35]. Spores of *B. anthracis* cause cutaneous, gastrointestinal, inhalational, and injection anthrax via the production of anthrax toxins and the formation of a poly‐γ‐d‐glutamic acid capsule, which protects the bacteria from phagocytosis and immune surveillance [36].

Biofilm is defined as a bacterial colony with a self‐produced matrix of extracellular polymeric substances that protects the bacterial cells from unfavorable external influences, such as temperature changes, dehydration, and biocides [21]. Bacterial biofilms are usually pathogenic, and it has been estimated that up to 80% of microbial infections in humans, including endocarditis, cystic fibrosis, periodontitis, rhinosinusitis, osteomyelitis, nonhealing chronic wounds, meningitis, kidney infections, and prosthesis and implantable device‐related infections, are associated with biofilm formation [37]. Many bacteria can form biofilms, with the most common ones being *E. coli*, *Enterococcus faecalis*, *Staphylococcus aureus*, *Staphylococcus epidermidis*, *Streptococcus viridans*, *Klebsiella pneumoniae*, *Proteus mirabilis*, and *Pseudomonas aeruginosa* [38]. Notably, *S. aureus* and *S. epidermidis* are estimated to cause approximately 50% of prosthetic heart valve infections, 70% of catheter biofilm infections, and 80% of bloodstream infections [38]. Unfortunately, the use of antibiotics alone is ineffective in treating biofilm‐related infections. This is because biofilms can delay or prevent the penetration of antibiotics [39], acquire resistance via horizontal gene transfer [40], and use multidrug efflux pumps to pump antibiotic agents out of the maturing biofilms and into the extracellular matrix [41]. In addition, biofilms can activate the innate immune system via secretion of C‐di‐NMPs, which induce an immune response through STING and subsequently activate type 1 IFNs [42].

### Via metabolic molecules

**Figure 2:**
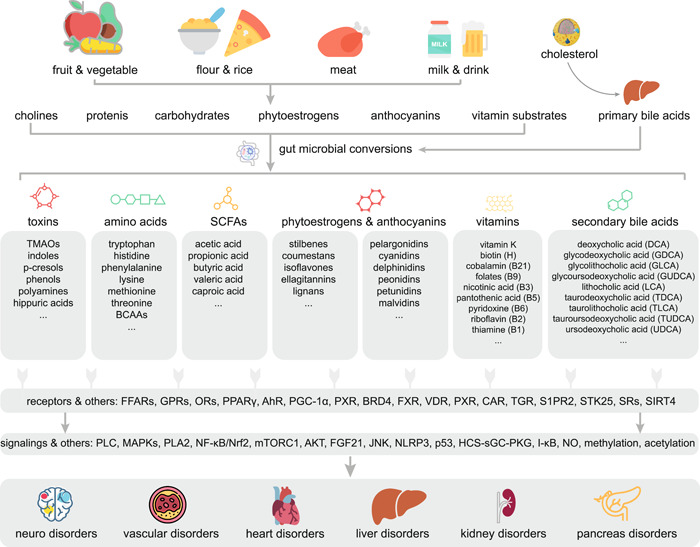
Gut microbial‐related metabolites that affect human health. Gut microbes are involved in the biosynthesis and biotransformation of a series of bioactive metabolites that can act as substrates and signaling molecules, contributing to normal human physiological functions or eliciting complex diseases. Specific classes of microbiota related metabolic molecules mainly include short‐chain fatty acids (SCFAs), amino acids, vitamins, bile acids, toxins, anthocyanins, and phytoestrogens

Gut microbes are involved in the biosynthesis and biotransformation of a series of bioactive metabolites that can act as substrates and signaling molecules, contributing to normal human physiological functions or eliciting complex diseases [13]. Specific classes of microbiota related metabolic molecules mainly include short‐chain fatty acids (SCFAs) [43], amino acids (AAs) [44], vitamins [45], bile acids (BAs) [46], toxins [47], anthocyanins [48], and phytoestrogens [49] (Figure 2).

SCFAs can be biosynthesized by gut microbes from the colon via fermentation of carbohydrates (e.g., glucose, starch, and fiber) or AAs (e.g., lysine, arginine, glycine, leucine, valine, and isoleucine) [50]. Gut microbial‐derived SCFAs mainly include acetic, propionic, butyric, valeric, and caproic acids. Notably, acetic, propionic, and butyric acids account for more than 95% of the total SCFAs and are present at a molar ratio of approximately 60:20:20 in the human gut [51]. Biosynthesis of acetate mainly relies on microbial genes encode phosphotransacetylase or acetate kinase [52]. For propionate, genes involved in succinate pathway, acrylate pathway, and propanediol pathway are essential [53]. For butyrate biosynthesis, pyruvate pathway, 4‐aminobutyrate pathway, glutarate pathway, and lysine pathway have been characterized [54]. Well‐known butyrate producers include a wide range of species that mainly belong to the Firmicutes phylum, including *Faecalibacterium prausnitzii*, *Eubacterium* spp., *Coprococcus* spp., and *Roseburia* spp. mediated by butyrate kinase or butyryl CoA:acetate CoA transferase [55]. The genome of *Bifidobacterium spp*. harbors several carbohydrases, which allow them to participate in the production of acetate and lactate during nondigestible carbohydrate breakdown. However, laboratory studies have shown that the ability of *Bifidobacterium* spp. to produce SCFAs is highly strain‐dependent owing to the variety in gene content [56, 57]. In addition, *Akkermansia muciniphila* produces SCFAs by mucin degradation and is a beneficial bacterium that induces the secretion of anti‐inflammatory cytokines and enhances the intestinal mucosal barriers [58].

SCFAs are important fuels for the human host, and they control the luminal pH. In addition, SCFAs are closely related to human health and disease. The production of the SCFA butyrate by the gut is associated with improved insulin response after an oral glucose tolerance test, whereas abnormalities in the production or absorption of propionate are related to an increased risk of T2D [59]. The mechanisms underlying the important roles of SCFAs in human physiological processes may rely on their signaling capacities in activating the free fatty acid receptors (FFARs, FFAR2, and FFAR3), G protein‐coupled receptors (GPR109A and GPR42), olfactory receptors (OR51E1 and OR51E2), peroxisome proliferator‐activated receptor‐γ and the aryl hydrocarbon receptor (AhR) [60, 61, 62]. Activation of these receptors by acetic, propionic, and butyric acids can further result in the activation of signaling cascades, including phospholipase C, mitogen‐activated protein kinase (MAPK), phospholipase A2, and nuclear factor‐κB (NF‐κB) pathways. These pathways are known to be involved in the etiology of various complex diseases owing to their functional roles in regulating satiety, energy harvesting, fat storage, adipose inflammation, and neuro system [60, 61, 62]. In addition, intracellular SCFAs can influence acetylation and deacetylation of histones (mainly 3 and 4), which mainly occurs on the epsilon amino groups of lysine residues on the N‐terminal tails. This increases the accessibility of the transcriptional machinery to promote gene transcription. This process occurs by inhibiting the activity of histone deacetylases (HDACs), resulting in more transcriptionally active chromatin, or by increasing the activity of histone acetyltransferases, thereby stimulating acetylation. HDACs are involved in a range of complex diseases, including colorectal cancer and Alzheimer's disease [61]. Butyrate, propionate, and acetate inhibit HDACs, with butyrate being the most potent [63]. Therefore, SCFAs produced by gut microbes may act as modulators of complex diseases.

AAs can be produced by gut microbes via digestion of food proteins or through de novo biosynthesis. Importantly, all the nine human essential AAs, including histidine, lysine, methionine, phenylalanine, threonine, tryptophan, isoleucine, leucine, and valine can be biosynthesized by the gut microbiota [64], through a large group of oxaloacetate/aspartate AAs biosynthesis genes [65]. Studies have shown that manipulating microbial genomes, for example, *fldC* in *Clostridium sporogenes*, could change the human blood aromatic AAs [66]. In addition to the roles as substrate for protein assembly and fermentation of SCFAs, deficiency of AAs is related to human disorders. Among them, tryptophan is the most chemically complex AA, which is associated with both host‐ and microbiota‐dominated pathways. Tryptophan decarboxylases have been observed in several bacterial genomes, including *Lactobacillus* spp., *Peptostreptococcus* spp., *Bacteroides* spp., and *Bifidobacterium* spp., which play an important role in the conversion of tryptophan to tryptamine and indole derivatives [67, 68]. The downstream metabolites can be sensed by different host intestinal receptors and thereby participate in regulating a variety of molecular pathways. These receptors include GPR35, AhR, serotonin receptors (5‐HT4R and 5‐HT3R), peroxisome proliferator‐activated receptor‐γ coactivator 1α (PGC‐1α), and pregnane X receptor (PXR) that are associated with brain, skeletal muscle, pancreas, and kidney disorders [50]. Histidine may impair insulin signaling in T2D through activation of the p38γ–p62–mTORC1 pathway [69]. Phenylalanine can be derived from dopamine and is associated with nervous system disorders such as Parkinson's disease [70]. Lysine, methionine, and threonine are derived from the oxaloacetate/aspartate AA biosynthesis pathway that is involved in insulin secretion and glucose metabolism via mitochondrial sirtuin 4 (SIRT4) [71], fibroblast growth factor 21 [72], and serine/threonine‐protein kinase 25 [73], respectively. In addition, leucine, isoleucine, and valine are branched‐chain amino acids associated with insulin resistance and glucose intolerance; however, the mechanism is unclear [74].

Toxins can be generated by gut microbes from various substrates, including AAs and choline class compounds. Protein‐bound uraemic toxins such as TMAO, indole, p‐cresol, phenol, and their sulfates and glucuronides, polyamines, as well as hippuric acid are derived from AAs by gut microbes [47]. Microbial genes that encode choline‐TMA lyase (*cutC/D*), carnitine monooxygenase, betaine reductase, and TMAO reductase are responsible for TMAO and derivatives [75]. The production of AAs derived uremic toxins such as indole, p‐cresol, and phenol largely depends on the gene content across different taxa [75]. For example, the gene coding tryptophanases presents differently in *Bacteroides* species and therefore, only certain *Bacteroides* spp. produce indoxyl sulfate [76]. Such toxins can further induce chronic kidney disease and CVD through the NF‐κB, MAPK, and Jun N‐terminal kinase pathways, thereby initiating the transcription of proinflammatory cytokines and adhesion molecules leading to inflammation and oxidative stress [47, 77]. Toxins derived from choline class compounds chiefly include TMAO and its derivatives, which have several roles in CVD, and probably act via MAPK and NF‐κB signaling [78], as well as via NLRP3 inflammasome [79], leading to inflammation. In addition, Gram‐negative bacteria, primarily from the *Bacteroidales* order [80], can biosynthesize the toxin LPS, which plays a role in coronary artery disease through the NF‐κB pathway [81].

Vitamins are essential human nutrients that must be obtained from exogenous sources, including food and the gut microbiota. The gut microbiota mainly synthesizes vitamin K and most of the water‐soluble B vitamins, such as biotin (H), cobalamin (B12), folate (B9), nicotinic acid (B3), pantothenic acid (B5), pyridoxine (B6), riboflavin (B2), and thiamine (B1), which are produced by 40%–65% of human gut bacteria [82]. The potential microbial pathways that is responsible for B vitamins biosynthesis have been introduced recently [83]. It has been estimated that up to half of the daily vitamin K requirement is provided by the gut microbiota (e.g., *Bacteroides*, *Bifidobacterium*, and *Enterococcus*) [84]. Notably, the production of vitamin K and water‐soluble B vitamins isoforms vary across strains with different enzymes [85]. Vitamin K plays a key role in blood clotting and building bones, as both prothrombin and osteocalcin require this vitamin [86]. In addition, vitamin K can regulate the NF‐κB/Nrf2 pathway via activation of Gla proteins to influence vascular inflammation in T2D [87]. Furthermore, B vitamins have transcriptional regulatory roles. For example, biotin acts via the holocarboxylase synthetase‐soluble guanylate cyclase‐cGMP‐dependent protein kinase (PKG) pathway [88], pyridoxine, cobalamin, and pantothenic acid act via Nrf2 [89, 90, 91], folate acts via interaction with bromodomain‐containing protein 4 and the folate pathway enzyme methylenetetrahydrofolate dehydrogenase, cyclohydrolase, and formyltetrahydrofolate synthetase 1 [92], nicotinic acid acts via G protein‐coupled receptor 109 [93], riboflavin acts via DNA methylation [94] and thiamine via p53 [95].

BAs are amphipathic steroids that are synthesized from cholesterol in the liver, referred to as primary BAs. Primary BAs can be reabsorbed from the small intestine and further be structurally modified by colonic microbes to form secondary BAs [96]. This process is mediated by 7α/β‐dehydroxylation enzymes. A recent study has characterized hundreds of microbial genetic structural variation associations to the human plasma BAs, but the functionalities of majority of those structural variation were unknown [97]. Microbial structural variants (SVs) are highly variable segments of bacterial genomes, including presence/absence (deletion SVs) and copy number variations (variable SVs) that have been defined in recent years based on metagenomic sequencing data [98]. In addition to their roles in bile formation, facilitating the absorption of intestinal lipid and fat‐soluble vitamins, maintenance of cholesterol homeostasis, and antimicrobial actions in the small intestine [99], several other functions of BAs have been discovered in the past two decades [46, 100]. It has been established that BAs exert hormone‐like actions to control glucose, lipid, and energy metabolism modulate immune functions and cellular proliferation and control detoxification reactions [46, 100]. The actions of BAs are mediated through activation of nuclear receptors, that is, the established BA receptor farnesoid X receptor as well as vitamin D receptor, PXR, constitutive androstane receptor as well as membrane‐bound receptors, such as Takeda G protein‐coupled receptor 5 and sphingosine‐1‐phosphate receptor 2 [101]. Importantly, differently structured primary and secondary BAs that are present within a certain type and between different types show wide variability in their capacities to exert classical as well as signaling functions [102]. This appears to be of physiological relevance since remarkable interindividual variations in plasma BA concentration and composition have been reported in several human cohorts associated with liver fat content [103], fatty liver disease [104], T2D [105], as well as various plasma lipid parameters [103].

Anthocyanins are flavones containing a phenolic structure that are widely distributed in plant vacuoles and demonstrate pH‐dependent color. Anthocyanins are known for their possible health benefits in preventing various conditions, including CVD, cancer and neurodegenerative disorders, and improving visual and brain functions [48]. Pelargonidin, cyanidin, delphinidin, peonidin, petunidin, and malvidin are the common anthocyanins occurring naturally in food [75, 106]. Notably, the prebiotic effects of anthocyanins rely on microbial modulations. For instance, catabolism of the anthocyanin cyanidin‐3‐glucoside in the gut microbiome results in the production of phenolic compounds, including protocatechuic acid, vanillic acid, phloroglucinaldehyde, and ferulic acid, which have an effect on oxidative stress and inflammation in the gut via activation of the Nrf2, MAPK, and NF‐κB pathways [106]. The microbiota anthocyanin metabolite gallic acid (GA) has been shown to increase the levels of nitric oxide by increasing the phosphorylation of endothelial nitric oxide synthase [107]. GA also inhibits the angiotensin‐I converting enzyme, leading to a reduction in blood pressure [108].

Phytoestrogens are nonsteroidal secondary metabolites of plants with unique diphenolic structures that include different classes of chemical compounds such as stilbenes, coumestans, isoflavones, ellagitannins, and lignans [49]. Phytoestrogens can be found in our daily diet and exhibit various physicochemical and biological effects, including antioxidative, antibacterial, anti‐inflammatory, anticarcinogenic, and cardioprotective effects [109]. Similar to anthocyanins, phytoestrogens preferentially bind to estrogen receptors (ERs) with weak affinity [110]. However, the variants of phytoestrogens transformed by the gut microbiome through novel enzymatic reactions can substantially enhance their bioactivities. The gut microbiome can transform phytoestrogens into molecules, such as equol, enterolactone, and enterodiol [111]. Equol can bind to the nuclear ERs expressed in many regions of the brain to improve the development of the cerebellum [112]. Both enterolactone and enterodiol can alleviate the effect of peripheral blood lymphocytes activated by LPSs, which further leads to inhibitory‐κB degradation and NF‐κB activation, thereby resulting in the production of TNF‐α [113].

### Via interactions with drugs

**Figure 3:**
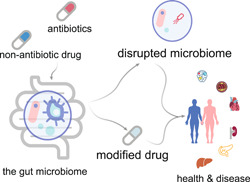
Microbial interactions with drugs. The gut microbiome can influence human health and disease through bidirectional interactions with drugs. On one hand, antibiotics can kill most of the gut bacteria that play important roles in maintaining the metabolic health of the host via a series of mechanisms. On the other hand, commonly used nonantibiotic drugs can be influenced by the gut microbiome via an enzymatic transformation that changes their bioavailability, bioactivity, or toxicity

The gut microbiome can influence human health and disease through bidirectional interactions with drugs (Figure 3). On one hand, antibiotics can kill most of the gut bacterial species that play important roles in maintaining the metabolic health of the host via a series of mechanisms [114, 115]. For instance, penicillin works by attacking the cell wall of bacteria to prevent them from synthesizing peptidoglycan, which provides strength to the wall required for survival in the human body [116]. Quinolones target DNA gyrase, an important enzyme that helps unwind DNA for replication to prevent bacterial multiplication [117]. Tetracycline prevents key molecules from binding to selected sites on ribosomes to stop asexual reproduction [118]. The antituberculosis antibiotics belonging to the rifamycin group exert a similar effect by inhibiting the synthesis of RNA [119].

In contrast, commonly used nonantibiotic drugs can be influenced by the gut microbiome via enzymatic transformation that changes their bioavailability, bioactivity, or toxicity [120]. A recent study conducted in vitro tests to assess the ability of 76 bacterial strains from the human gut, representing 68 species from the main bacterial taxonomic groupings, to metabolize 271 drugs. The drugs were chosen to include a diverse group based on factors, such as molecular structure or effect on the body. The study reported that 176 drugs demonstrated a substantial metabolic change caused by at least one bacterial strain, which resulted in reduced levels of the active drug molecule in the bacteria [121]. These results state the possibility that most drugs are modified by the microbiota, and such tests could prove useful during drug selection by isolating the agents that would probably be deactivated by specific gut microbes.

## TOOLS FOR DECODING THE GUT MICROBIAL FUNCTIONALITIES

Based on high‐throughput next‐generation sequencing, which provides targeted or the whole microbial genomes, a series of bioinformatics tools have been developed to decode the microbial DNA sequence and predict their functionalities. In general, such tools can be divided into three categories based on their theories, namely, taxonomic marker gene‐based indirect prediction, gene homology‐based direct prediction, and sequence similarity‐based de novo prediction.

### Taxonomic marker gene‐based indirect prediction

Amplicon‐based sequencing of marker genes like 16S ribosomal RNA is a powerful tool to assess and compare the structure of microbial communities within or between samples. However, insights into the functional capabilities of the gut microbiome are limited because the sequence information is only derived from specific genomic regions. Nevertheless, researchers often infer functions of uncultured organisms from their cultured counterparts, as a clade's core genome consists of genes, which its members can be expected to carry with a high probability. Thus, functions encoded in the genome of an organism may partially be predicted based on the functions encoded in closely related and well‐annotated genomes. Based on this theory, tools including PICRUSt2 [122], Tax4Fun2 [123], BugBase [124], and Piphillin [125] have been developed to profile functional components of the gut microbiome based on taxonomy information (Table 1).

**Table 1:** Summary of taxonomic marker gene‐based functional prediction tools

Tool	Description	Taxa database	Functional gene database	Functional pathway database	Link
PICRUSt2	Ancestral‐state reconstruction based	ASVs derived from IMG	KEGG, EC number	MetaCyc	https://github.com/picrust/picrust2
Tax4Fun2	16S rRNA gene BLAST based	Ref100NR derived from NCBI RefSeq	KEGG		https://github.com/bwemheu/Tax4Fun2
Bugbase	PICRUSt based but with clinical phenotypes	Greengenes	KEGG		https://bugbase.cs.umn.edu/index.html
Piphillin	Nearest‐neighbor matching based	Manully created	KEGG		https://piphillin.secondgenome.com

Abbreviation: KEGG, Kyoto Encyclopedia of Genes and Genomes.

PICRUSt2 [122] uses an extended ancestral‐state reconstruction algorithm based on IMG [126] to predict the gene families present and subsequently combines the gene families by a weighting method to estimate the composite metagenome. Tax4Fun2 [123] relies on the identification of the nearest neighbor with Ref100NR and generates Kyoto Encyclopedia of Genes and Genomes (KEGG) [127] outputs with normalizations and linear combinations. Piphillin [125] uses global nearest neighbor matching to generate operational taxonomic unit abundance tables that are independent of any proposed phylogenetic tree and further links to the most updated KEGG to profile the functional components. BugBase [124] utilizes a phylogenetic approach to predict genomic content based on 16S and biologically interpretable phenotypes such as oxygen tolerance, Gram staining, and pathogenic potential with existing knowledge. As the predictive power of the aforementioned tools chiefly relies on the functional information derived from the available genomes, recent progress in the construction of metagenomic‐assembled genomes [128] is likely to enhance the accuracy of functional inferences after incorporation.

### Gene homology‐based direct prediction

Compared with the taxonomic marker gene‐based indirect prediction, massive sequencing reads generated by shotgun metagenomic/metatranscriptomic sequencing (MGS) that cover the entire genomes rather than marker genes can result in more accurate prediction of the gut microbial functionalities via directly mapping reads against well‐annotated gene databases. The commonly used tools for functional prediction of MGS include HUMAnN3 [129], MEGAN [130], ShotMAP [131], and gutSMASH [132] (Table 2). HUMAnN3 [129] generates species‐level gene abundances based on UniRef [133] and further assigns them to MetaCyc pathways [134]. MEGAN [130] profiles microbial functionalities based on SEED [135], eggNOG [136], and KEGG. ShotMAP [131] translates reads into predicted open reading frames and further searches the SFams [137] protein family database. GutSMASH [132] mines primary specialized metabolic gene clusters that are responsible for the biosynthesis of various metabolites in the human gut microbiome with the taxonomic resolution based on the KnownClusterBlast and ClusterBlast databases.

**Table 2:** Summary of gene homology‐based functional prediction tools

Tool	Description	Taxa database	Functional gene database	Functional pathway database	Link
HUMAnN3	Profiling gene and pathway abundances with species resolution	UniRef	UniRef	MetaCyc	https://huttenhower.sph.harvard.edu/humann
MEGAN	Cross‐platform software for taxonomic level functionalities	NCBI taxonomy	SEED, eggNOG, KEGG, Pro2GO		https://github.com/danielhuson/megan-ce
ShotMAP	ORF finding based homology inference and family classification		KEGG, FIGfams, SFams		https://github.com/sharpton/shotmap
gutSMASH	Profiling pathways for primary metabolites with strain resolution	1621 Microbial genomes	MGCs	MGCs‐derived pathways	https://gutsmash.bioinformatics.nl/help.html#Validation

Abbreviations: KEGG, Kyoto Encyclopedia of Genes and Genomes; MGC, metabolic gene clusters; NCBI, National Center for Biotechnology Information; ORF, open reading frame.

### Sequence‐based de novo prediction

The current microbial genomic annotation pipelines are based on the principle of sequence similarity with existing databases, such as UniRef [133], MetaCyc [134], SEED [135], eggNOG [136], and KEGG [127]. Currently, only approximately 60% of the microbiome genomes can be annotated [128] with a homolog‐based approach, and nearly half of the microbial functionalities remain a mystery. In addition, genetic polymorphisms arise rapidly through de novo mutations (e.g., single‐nucleotide variations), which could have regulatory effects on gene expression and functions. Notably, a prevailing belief across modern molecular biology research is that a gene sequence defines the structure of the gene product and this structure, in turn, designates a unique function [138]. In other words, even with 99% similarity between the sequences of two genes, their functionalities may be completely different due to structural differences caused by variations in the remaining 1%. Thus, predicting the functionality of microbial genes based on the structure of the end product, for example, protein structure, can be a promising approach. Recently, a novel machine learning approach named AlphaFold has been developed to predict protein structures with atomic accuracy even in cases where the homologous protein structure is not known [139]. AlphaFold incorporates physical and biological knowledge regarding the protein structure and leverages multisequence alignments into the design of the deep learning algorithm. It does not impose known rules of protein biophysics or mimic the physical process of protein folding. Instead, AlphaFold performs purely geometric refinements learned from repeated attempts to predict protein structures. Thus, it may sweep the field of decoding microbial functionality in a novel manner.

However, before utilizing shotgun metagenomic sequencing data for decoding strain‐level microbial functionalities, the binning of short reads is considered a crucial step. There are two types of binning approaches, including reference‐dependent and independent binning. Reference‐dependent approach basically maps reads against a database of existing microbial reference genomes using tools such as bowtie2 [140]. But the main drawback is that it lacks the ability to characterize unknown microbial genomes. Reference independent approach is an unsupervised method to cluster contigs into individual genome bins without the assistance of any reference databases. The performance of various tools for metagenomic genome binning has been evaluated recently [141], and highlighted that most genome binning tools performed well for unique strains but reconstructing common strains still is a substantial challenge for all genome binning tools [141]. This may be due to the fact that common strains shared similar genomes that cannot be discriminated easily. Nevertheless, advances in the long‐read sequencing may facilitate de novo binning [142].

## TECHNIQUES FOR VALIDATING MICROBIAL FUNCTIONALITIES

**Figure 4:**
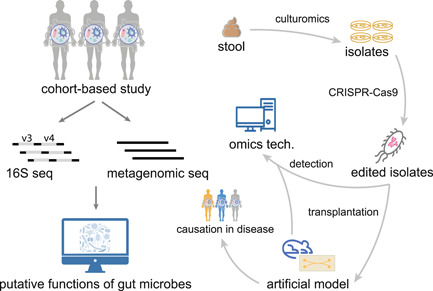
Verifying microbial functionalities with state‐of‐the‐art techniques. Many gut microbes have been found to be associated with various diseases in human participant‐based studies. Although the potential functional role of the gut microbes can be predicted via various bioinformatics tools, the proposed putative functionalities of gut microbes of interest lack functional validation. Thus, taking advantage of state‐of‐the‐art techniques such as culturomics, genome editing, novel models, as well as multiomics may further strengthen our understanding of their functionalities and entirely develop the gut microbiome‐based personalized medicine

In silico approaches have identified hundreds of microbes through association‐based theory, which are likely to be important in human health and disease. However, the proposed putative functionalities of gut microbes of interest lack functional validation. Thus, taking advantage of state‐of‐the‐art techniques such as culturomics, genome editing, novel models, as well as multiomics may further strengthen our understanding of their functionalities and entirely develop the gut microbiome‐based personalized medicine (Figure 4).

### Culturomics

Sequencing the gut microbiome highlighted that most bacteria in the gut remain uncultured and revealed the functional importance of specific gut microbes. However, the technique might be associated with bias in DNA extraction protocols, bioinformatics tools, as well as minority microbial populations. Consequently, culturomics was developed to culture and identify unknown bacteria that inhabit the human gut for direct functional validation and clinical application. Culturomics is a culturing approach that uses multiple culture conditions, mass spectrometry(, and a sequencing approach to identify bacterial species [143]. The first step in culturomics is to enable the provision of multiple culture conditions and promote the growth of fastidious bacteria from the human gut. This is achieved by improving the culture media to promote the growth of minority populations. Next, mass spectrometry is performed for the rapid identification of microbial species, which relies on the comparison of the protein mass spectra of the isolate with the most updated database. Following this, 16S and whole‐genome sequencing are applied to confirm the new taxa by comparing the existing microbial genomes recovered from humans. The application of culturomics has resulted in thousands of bacterial isolates, and a substantial proportion of them have been considered novel species/strains [144, 145, 146, 147]. Such resources allow us to test the association‐based functional hypotheses directly with isolates in mechanistic studies when coupled with in vitro and animal models or clinical applications such as microbiota transplantation and microbial editing with CRISPR‐Cas9.

### Genome editing

It has been shown that structure variations widely exist in the gut microbial genomes [11]. Besides, single nucleotide polymorphism (SNP) level phylogenetic analysis of worldwide metagenomic samples showed remarkable within‐species genetic variability [128]. Variations observed in the genomes of microbial strains from the same species may vary in their functionalities. To understand the role of genomic variations, modifying genomes of microbial isolates with genome editing tools such as CRISPR‐Cas9 [148] is a promising approach to test genetic regulations of microbial functionalities.

### Humanized animal and organ‐on‐chip models

Human participants cannot be directly subjected to verification of unpredictable functional roles of the gut microbiome in health and disease due to ethical issues. Consequently, novel in vivo and in vitro models such as humanized animal and organ‐on‐chip technology have emerged as the next‐generation disease and drug models [149, 150]. Humanized animal models are animal models with human‐like phenotypes obtained by editing the animal genome or inducing external perturbations. For instance, a mouse model with a human‐like BA pool has been generated by knocking out the Cyp2c70 gene with CRISPR/Cas9 [148], which might be a powerful tool to reveal the effects of the gut microbiota on BA metabolism and CVD. In the organ‐on‐a‐chip model, human induced pluripotent stem cells can be differentiated to obtain different tissue and cell types that can be used to construct organs‐on‐chips. In particular, organs‐on‐a‐chip such as gut‐on‐a‐chip and liver‐on‐a‐chip would be very interesting to investigate microbe‐intestine and host–microbe metabolic interactions.

### Multiomics

Instead of accessing microbial functionalities via genomes, generating various types of omics datasets such as metabolomics, proteomics, and transcriptomics, and further linking them to the gut microbiome may enhance our understanding regarding the importance of gut microbes in complex diseases progressing from taxonomical association to potential functionality. However, challenges persist in both proteomics and metabolomics that prohibit further exploration of the functionality of gut microbes. Although the traditional targeted approach can result in accurate identification and quantification of individual metabolites or proteins. But its low throughput and relatively high cost make it less suitable for application in large cohort studies. Untargeted approaches by innovative tandem mass spectrometry approaches can profile thousands of molecules after a single injection; however, functional annotation and quantification remain a bottleneck in this approach. Although community guidelines for metabolite identification were published over a decade ago, adoption of the recommended standards has been limited [151]. Developing targeted extraction/identification protocols for specific metabolite and protein classes might be a promising approach to resolve these issues. In addition, with a better understanding of enzymatic functions, the gap in knowledge regarding unknown metabolites and proteins is reducing. Knowledge of metabolic reactions should promote the development of more powerful identification tools. For transcriptomics, a major challenge would be the isolation of microbial RNA, as the fecal samples are complex which makes it hard to get high‐quality microbial RNA. However, the development of a single bacterial sequencing technology might be a good solution.

## FUTURE PERSPECTIVES

Nowadays, microbiome studies are mainly focused on the abundance of microbial taxa and functional genes. However, we have to bear in mind that the abundance of gut microbes generated by bioinformatic pipelines from sequencing data cannot really reflect the real density (absolute abundance) of microbial organisms in the human gut. If the microbial load varies substantially between samples, relative profiling will hamper attempts to link microbiome features to quantitative data such as metabolite concentrations [152]. As a cause, the quantitative microbiome profiling method that combines microbial cell count using flow cytometry with fecal microbiome sequencing data has been developed recently [153], which provides more power in assessing microbial variation within and between individuals. However, drawbacks still exist as the method is lab skill dependent in which a single measurement does not estimate the equilibrium abundance well. Besides this, it is expensive and time‐consuming and not suitable for big cohort‐based studies. Thus, future improvements in this technique are needed.

Apart from the importance of variations of the gut microbial genes in copy numbers, studying variations in microbial genomes is another essential direction to go. Like the human genome, SNPs, SVs (e.g., insertion and deletion), mobile genetic elements (e.g., bacteriophages and transposable elements) in the microbial DNA sequences may also be important for microbial functionalities and related to human diseases [11, 154]. In the past decades, genetic regulation of microbial functionalities mainly focused on the limited number of well‐known genes. Yet, systematic microbial genome‐wide association (e.g., SNPs and SVs) to physiology measurements or metabolite concentrations generated with omics techniques is still absent, which may reveal novel knowledge regarding the functional role of the human gut microbiome in host health and disease with a much higher resolution than microbial abundances. However, many challenges remain in both bioinformatics (e.g., problems in genome binning that have been discussed in the above sections) and statistics (e.g., millions of SNPs in the microbial genomes increase the number of statistical tests, which will have a negative effect on detection power). The employment of long‐read sequencing and common variants might be a potential direction to explore at the early stage.

## CONCLUSIONS

The effects of gut microbiota on the host are mainly mediated by microbial virulence factors, metabolic molecules, and bidirectional interaction with drugs. We have highlighted the functional potential of gut microbes in human health and disease and summarized the associated molecular mechanisms. In addition, bioinformatics tools have been introduced that can be applied to decode the functionalities from microbial genomes, which might be helpful for researchers to prioritize them for a specific purpose. Finally, we highlighted the importance of culturomics and genome editing, multiomics, as well as novel models for functional verification and stated the possibilities of modulating the gut microbiome to improve human health.

## CONFLICTS OF INTEREST

The authors declare no conflicts of interest.

## AUTHOR CONTRIBUTIONS

Lianmin Chen contributed to conceptualization and writing. Yifeng Wang, Quanbin Dong, Shixian Hu, Huayiyang Zou, Tingting Wu, Jing Shi, Huayiyang Zou, Yanhui Sheng, Wei Sun, and Xiangqing Kong contributed to the discussion of the content. All authors read and approved the final manuscript.

## Data Availability

This manuscript does not generate any script and data. Supporting Information (graphical abstract, slides, videos, Chinese translated version, and update materials) are available online DOI or http://www.imeta.science
